# Stramonium Poisoning—Antidotal Power of Opium

**Published:** 1871-01

**Authors:** 


STRAMONIUM POISONING—ANTIDOTAL POWER OF OPIUM.—A case of poisoning by stramonium, in which opium appears to have been useful as an antidote, is reported by Dr. Cheney in another portion of the *Journal.* The value of opium and belladonna as antidotes one to another, is not yet determined. Stramonium is so nearly allied to the latter, as to leave little room for doubt that it holds the same relation to opium. Children are rather fond of eating the seeds of stramonium, but the effects are rarely fatal. Many cases are reported in support of the mutual antidotal power of opium and belladonna; but the difficulty is to determine whether death would have occurred without the antidote. Persons recover from enormous doses of either narcotic, under ordinary treatment, or in some instances without interference. The nearest approach to death that we have ever known to be followed by recovery, was in a case of poisoning from laudanum, in which the patient—a female who contemplated suicide—continued to sink after the free use of the stomach pump, cold affusions, and other means. For several hours she lay in a hopeless condition, the heart and lungs scarcely exhibiting any perceptible movement. But she revived after all this. Had belladonna been administered, we should undoubtedly have ascribed the result to its antidotal virtue. The subject is one of great interest, and is open to further investigation.—*Pacific Medical and Surgical Journal.*

